# Trends in Autism Spectrum-Related Motherhood Research: A Bibliometric Study

**DOI:** 10.3390/ejihpe13020036

**Published:** 2023-02-16

**Authors:** Osvaldo Hernández-González, Daniela González-Fernández, Rosario Spencer-Contreras, Raúl Tárraga-Mínguez, Viviana Ponce-Carrasco

**Affiliations:** 1 Faculty of Psychology and Institute of Humanistic Studies Juan Ignacio Molina, Universidad de Talca, Talca 3480094, Chile; 2 Faculty of Health Sciences, Universidad Autónoma de Chile, Santiago 7500912, Chile; 3 Faculty of Psychology, Universidad de Talca, Talca 3480094, Chile; 4 Department of Education and School Management, Faculty of Teacher Training, University of Valencia, 46010 Valencia, Spain

**Keywords:** autism, bibliometrics, mothers, mother–child interaction

## Abstract

Autism spectrum disorder (ASD) is a neurodevelopmental condition characterized by difficulties in socialization. The amount of scientific research results on motherhood related to ASD has grown exponentially; however, there are no bibliometric studies in this field. Objective: This article aimed to analyze scientific research on motherhood related to the autism spectrum published in WoS. Articles on motherhood related to ASD were retrieved from the Web of Science (WoS). The advanced search interface used was “Title of the article, Abstract, Keywords”. The analysis and visualization of the selected documents and their data were performed using a wide range of tools and software such as MS Excel (v16.0), VOS viewer (version 1.6.15), and R packages (Biblioshiny, version 2.0). A total of 1660 articles were included in this study. Most of the publications were original articles. The United States published the most significant number of articles among the countries identified. P.R. Hastings, M.M. Seltzer, and J. Van de Water were the main authors. *The Journal of Autism and Developmental Disorders* was the most productive and impactful journal. The main research topics were related to mental health and social support in the role of motherhood. This desk study provides researchers with a comprehensive understanding of ASD-related maternity research trends by evaluating relevant publications in recent decades. The results of this bibliometric analysis can serve as a basis and orientation for new studies.

## 1. Introduction

Autism spectrum disorder (ASD) is characterized by qualitative deficits in social interaction and communication, as well as restricted and repetitive interests and behaviors that begin in the first years of life [1]. Diagnostic evaluation of ASD can be performed from 18 to 24 months of age, since in this developmental stage, ASD symptoms can be distinguished from typical development and other neuropsychiatric pathologies [2]. The prevalence of this disorder has exponentially increased with diagnostic reports of one in fifty-nine people [3]. Disruptive behaviors inherent in ASD overlap with other medical conditions such as anxiety disorders [4], depression [5] or eating disorders [6]. This clinical overlap makes the socioemotional display of people with ASD even more problematic. The education of a child with ASD can have a significant impact on the psychological and social well-being of mothers [7,8].

The behavioral characteristics of people with ASDs imply substantive and early changes in the areas of socialization and, therefore, often require extensive care and permanent commitments from families [9,10]. Children and young people with ASD need continuous and timely support from mothers to develop early skills that allow them to socialize and tolerate the uncertainty of a constantly changing world [11]. The advent of a child with ASD can have a significant impact on the family life of mothers and/or responsible caregivers [12]. These parenting idiosyncrasies cause socially responsible mothers of children with ASD to report higher levels of stress [13]. There is a relationship between the problem behaviors of the child and the inclination of the mothers to seek social support, since their role is key to changing the course of the ASD towards a more adaptive trajectory of development [14]. Some studies address the interaction of mothers with their autistic children, highlighting the strengths and weaknesses of the maternal interaction style, as well as the long-term benefits of a positive relationship [15,16,17].

In this sense, it has been reported that the quality of life of mothers of children with ASD is lower compared to other types of population and this is related to characteristics of ASD, and parental and environmental variables [18]. A narrative review conducted by Mconachie and Diggle (2007) [11] highlighted that parent training contributes to enhancing the child’s communication, greater maternal knowledge about ASD, a more appropriate maternal communication style, and a reduction in maternal depression. Another systematic review, by Ilias et al. [19], highlighted that social support, severity of ASD symptoms, financial difficulty, parents’ perception and understanding of ASD, parental anxiety, and concerns about their children’s future constitute a constellation of variables that Zhang [20] predict stress in mothers of children with ASD. A recent meta-analysis by Kulasinghe et al. [21] was able to demonstrate that interventions that seek to promote appropriate and timely parenting styles to respond to the cognitive, emotional, and volitional demands of children with ASD had a positive effect on the mother–child relationship and a moderate impact in general stress of mothers. However, no bibliometric analysis specifically focused on ASD-related motherhood has been conducted to our knowledge. The bibliometric analysis involves the quantitative analysis of the research literature [22] to highlight documents, trends, countries, institutions, and authors with the most significant impact in any field of knowledge. To carry out this technique, the Web of Science (WOS) was used, which is a multidisciplinary database that is considered one of the main bibliographic sources of information. Applying this technique can complement previous reviews and, thus, provide us a more global and complete picture of motherhood and ASD. Because of this research gap, the present study aims to analyze the scientific research on ASD-related motherhood published in WoS. This could provide an overview of the topic and ideas for researchers to discover new lines and directions. It is also a valuable resource for physicians, clinical psychologists, and other health professionals who are less familiar with the field and want to learn more about it. The following research questions were raised: Q1: What are general characteristics of the maternity literature related to ASD? Q2: What is the trajectory of the annual publication? Q3: Who are the most productive authors? Q4: What are the most productive journals? Q5: What were the most cited documents and the main results of each of them? Q6: What are the main keywords, co-occurrence network, and trending topics? Q7: In the intellectual structure, what is the co-citation network? Q8: In the social structure, what are the collaboration networks of authors, institutions, and countries?

## 2. Materials and Methods

This study performed a bibliometric analysis of the academic literature published in the Web of Science (WoS) on ASD-related maternity research. Bibliometric analysis is a discipline that uses documentary review and measurement features (for example, mathematics and statistics) to explore the structures, features, and laws of science [23,24]. Due to its value in understanding the behavior of the specialized literature, the application of this technique in the field of ASD grows every day [25,26,27].

### 2.1. Database Selection and Literature Search

WoS is a collection of bibliographic references and journal citation databases highly respected for journal selection and quality. We selected it for data extraction on publications related to motherhood and ASD. In the search, the advanced search interface used was: “Title of the article, Abstract, Keywords”. Keywords for this study were derived from previous articles [28,29,30] and were selected under the supervision of a specialist with professional experience (20 years) in the area of bibliometrics. Search terms were strategically combined using Boolean operators: (“Autism Spectrum Disorder” OR “Autism”) AND (“maternity” OR “motherhood” OR “mothers” OR “mother-child interaction”). The entire period declared by WoS (publication years 1975–2021) was used for the search. The search was conducted on 13 May 2022. A search alert was also left open in WoS so as not to rule out relevant articles that could be published while the study was being carried out. This search alert was canceled on 13 May 2022. A total of 4348 articles potentially relevant to the study were retrieved.

### 2.2. Inclusion/Exclusion Criteria

The main inclusion criterion was that the articles directly studied motherhood in ASD. Language and time restrictions did not apply. Articles that used motherhood but did not directly link it to ASD were excluded; articles related to ASD that did not directly use any variable linked to motherhood were also excluded.

### 2.3. Preliminary Evaluation of the Search

**Figure 1:**
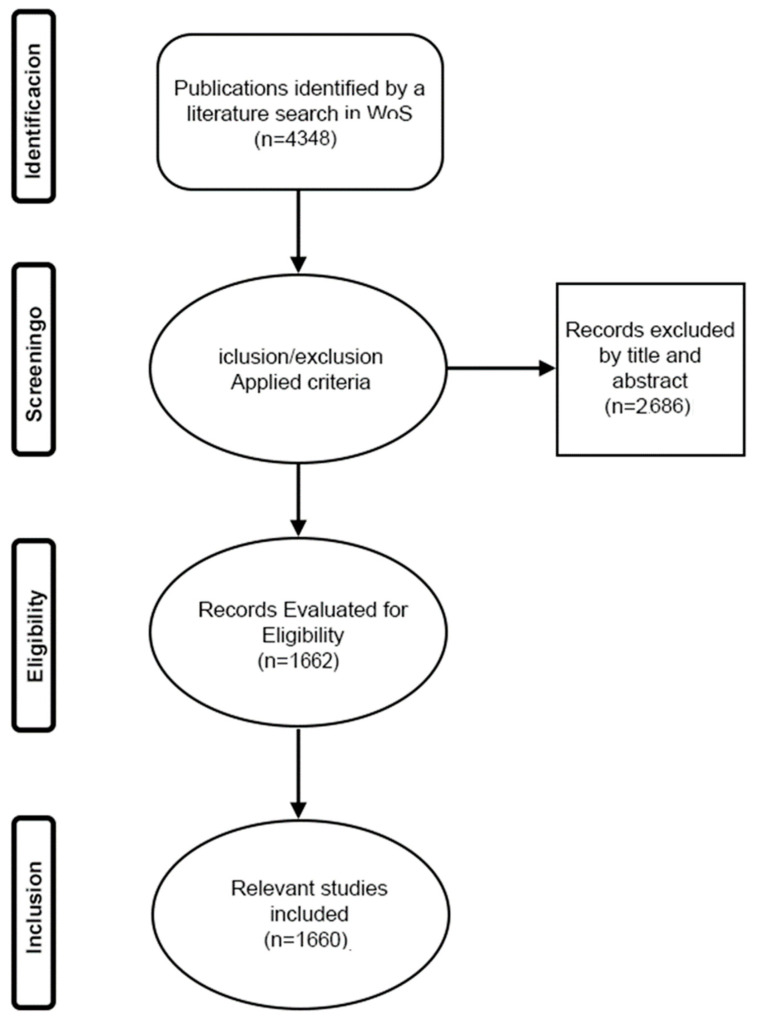
Flowcharts of four phases of the publication data extraction and filtering process.

The 4348 articles initially collected underwent a double-blind evaluation to eliminate bias in the application of criteria. In this sense, the relevance and eligibility of each record was evaluated, and 2686 irrelevant documents were excluded. This process was performed by two authors in parallel to eliminate selection bias. Double review resulted in 98.3% agreement (% of items matched with a probability of 1.0). Authors met via the Zoom platform and discussed articles where ratings differed. Once the details of each of the articles had been analyzed as a group, a consensus was reached on their inclusion or exclusion. Once the process was completed, 1660 records were selected for bibliometric analysis (see selection route in Figure 1). Finally, the records were exported to MS Excel. This entire preliminary evaluation process was completed on 31 June 2022.

### 2.4. Data Analysis and Visualization

Analysis and visualization of the selected documents and their data were performed using a wide range of tools and software such as MS Excel (v16.0), VOS viewer (version 1.6.15), and R packages (Biblioshiny, version 2.0). Through the operational virtues of these tools and software, a systematic description of the following indicators was constructed: general information characteristics; annual publication (behavior of all documents published in WoS); authors (top author performance metrics); Lotka’s law (the description of a quantitative relationship between authors and articles produced in a given field and in a period of time [31]. For example, in light of this law, most authors publish few articles, while a few produce the majority of them); journals (name and performance of the journal where the papers were published); Bradford’s law (Bradford’s law (1934) establishes the dispersion of scientific literature. For example, if the scientific journals are arranged in order of decreasing productivity of articles in a certain field of study, they can be divided into a nucleus of journals more particularly dedicated to the subject); primary documents (most cited papers in the field selected as the object of study); keywords (words generated from the titles of the cited documents); co-occurrence network (grouped interconnection of terms); thematic map (diagrams that represent the words, ideas, tasks, or other concepts linked and arranged radially around a keyword or a central idea); co-citation network (co-occurrence relationship that occurs when two items from the existing literature are cited together by a third party); and collaboration network of authors, institutions, and countries (a group of people, institutions, or governments that contribute intellectual work to a project with a common goal for the group).

### 2.5. Ethical Elements

Due to the documentary nature of this research, the approval of an ethics committee was not necessary.

## 3. Results

### 3.1. Output of General Information and Annual Publication

After the search and selection of papers, a total of 1660 records were identified. The main information of the sample was distributed as follows: DOCUMENT CONTENTS/keywords plus (2959), Author’s Keywords (3277); AUTHORS COLLABORATION/authors (5376), authors of single-authored docs (113), co-authors per doc (4.24), international co-authorships % (20.78); DOCUMENT TYPES/article (1416), book chapter (8), early access (38), proceedings paper (13), book review (11), correction (1), editorial material (12), letter (4), meeting abstract (54), note (1), review (100), book chapter review (2), and early access review (4). On the other hand, the number of annual publications in the last decades shows a development trend in research related to motherhood and ASD (see Table 1). It is possible to observe in Table 1 that the publications on the topic selected as the object of the documentary study appeared in the 1990s. The increase has been exponential. For example, in the first period, 6 documents were published, in the second period, 151 documents, in the third period, 1050 papers, and so far in the fourth period, 746 documents have been published. The most productive years were 2019, 2020, and 2021, showing that motherhood related to autism is a very current topic. Although 2022 was not complete (the selected months were January, February, March, April, May, June, July, August), it was selected to see and compare the publication rate of these first years.

### 3.2. Distribution of Authors

A total of 5376 authors contributed to the total number of publications. The main authors who published more than 10 articles on maternity variables associated with ASD are listed in Table 2. The most productive on this list (R.P. Hastings, M.M. Seltzer, and J. Van de Water) published more than 20 articles. Those same authors received the highest volume of international citations.

Lotka’s law refers to the distribution of authors according to their productivity [32]. Table 3 shows the approximation between the observed and expected values of the author’s productivity distribution. There is correspondence between the observed and expected scientific production, so Lotka’s Law is fulfilled.

### 3.3. Journal Distribution

A total of 580 journals have been receptive to maternity and ASD publications on WoS. Table 4 shows that *The Journal of Autism and Developmental Disorders* had the highest number of publications (169), followed by *Research in Autism Spectrum Disorders* (65) and *Autism* (57). The journal with the most significant impact turned out to be *The Journal of Autism and Developmental Disorders*.

(g- index) g number of publications receiving at least g^2^ citations (i.e., measure of impact) [33].

(1) $$g={\left(\frac{\propto -1}{\propto -2}\right)}^{\frac{\propto -1}{\propto }}T^{\frac{1}{\propto }}$$

Equation (1) provides the tool to calculate how many publications were cited at least h times.

(m index) The m index is defined as h/n, where h is the h-index and n is the number of years since the first published paper of the scientist.

On the other hand, in the opinion of Desai et al. [34], Bradford’s law indicates that citations on any research topic will cluster in such a way that approximately one third of the citations will occur within a small core of journals. Our results suggest that Core 1 encompasses 15 journals, and the list is headed by The *Journal of Autism and Developmental Disorders, Research in Autism Spectrum Disorders*, and *Autism* (see Table 5).

### 3.4. Analysis of the Main Documents

In Table 6, we can see the documents with the highest number of citations historically and their average number of citations per year (normalized). The first article is by Davis and Carter [35] and is entitled “Parenting Stress in Mothers and Fathers of Toddlers with Autism Spectrum Disorders: Associations with Child Characteristics” (601 citations). The results of this study indicate that parents reported high parental stress and that deficits/delays in children’s social engagement were associated with: (a) general parental stress, (b) problems in the parent–child relationship, and (c) the anguish of mothers and fathers. It is followed (479 citations) by the study by Cook et al. [36], entitled “Autism or atypical autism in the proximal duplication of 15q derived from the mother but not from the father”. In this study, we found that during the course of genotyping trios of AD-affected probands and their father, at the candidate positional locus D15S122, a proximal intrachromosomal duplication of 15q was detected by microsatellite analysis in a phenotypically normal mother. Among the most effective articles (more citations in fewer years) is the study by Malkova et al. [37], titled “Maternal immune activation produces offspring that show mouse versions of the three main symptoms of autism”, with 375 citations. Their results indicated that MIA (maternal immune activation) produces male offspring with poor social and communicative behavior, as well as high levels of repetitive behaviors, all of which are hallmarks of autism.

### 3.5. Keyword Analysis, Co-Occurrence Network and Thematic Map

**Figure 2:**
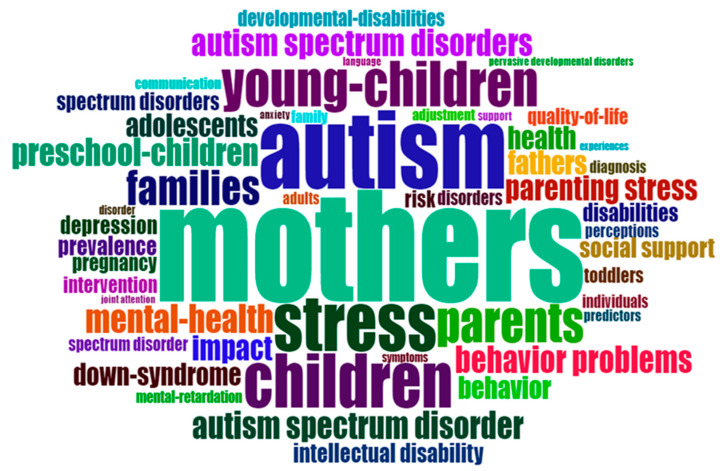
Word cloud. Note: The importance of a word is shown by font size and/or color.

Analyzing the keywords that the author places as orientation of the publications is a fundamental tool to investigate the relevance of a scientific topic [45]. In this sense, keywords are an orientation compass that helps to quickly identify the topic and focus of a scientific publication. The word cloud in Figure 2 shows the relevance of keywords in ASD maternity publications.

**Figure 3:**
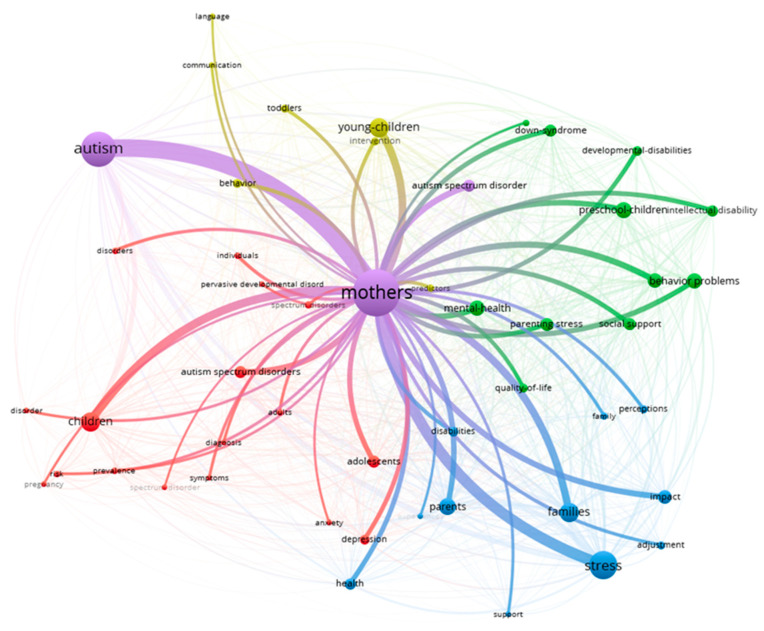
Keyword matching network (KCN).

This study also investigated the keyword co-occurrence network (KCN) to gain a global view of trends in childbearing and the autism spectrum. The KCN analysis presents the close relationship that occurs between the keywords in the literature, which provides an idea of the knowledge structure of the field [46]. This analysis provides an overview of the topic that goes far beyond the simple representation of the keywords, as it shows their connections (see Figure 3). It is possible to observe that the word with the highest representation, mothers, is connected to four thematic axes; for example, the green cluster is related to mental health and social support, the blue group is associated with the impact of autism on the family, the red cluster with a predominance of clinical semiology, and the yellow cluster with early childhood.

**Figure 4:**
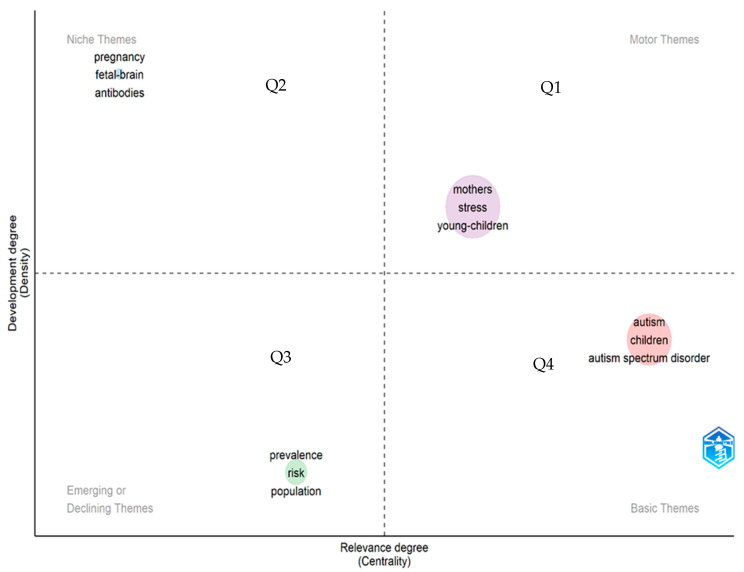
Thematic map. Note: Q1 contains the main theme, Q2 contains highly developed and specialized themes that link to the main theme, Q3 contains emerging or disappearing themes, Q4 consists of foundational and cross-curricular themes.

We also analyzed the thematic map of maternity studies related to ASD to obtain a holistic view of the current state of the field and its sustainability over time. These themes are characterized by two properties (density and centrality). Density is plotted on the vertical axis and centrality on the horizontal axis. Centrality is the degree of correlation between different themes, and density measures the cohesion between nodes [46]. The visualization of the themes helps us to understand if they are well developed and what their degree of importance is. In this sense, the greater the number of relationships that a node has with others in the thematic network, the greater its centrality and importance. In our case, we provide the thematic map of the field of maternity linked to ASD, divided into four quadrants (Q1 to Q4). For example, the upper right quadrant (Q1) represents driving issues, the lower right quadrant (Q4) represents underlying issues, the upper left quadrant (Q2) represents highly specialized issues, and the lower left quadrant (Q3) represents emerging issues or issues that disappear (see Figure 4).

### 3.6. Intellectual Structure: Co-Citation Network Analysis

**Figure 5:**
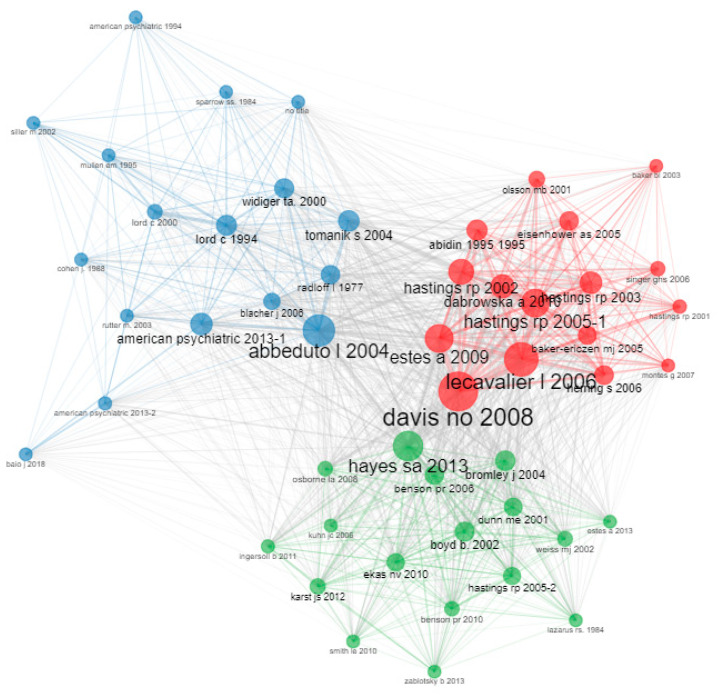
Co-citation network.

Regarding co-citation authors, according to Aria and Cuccurullo [47], closeness measures how many steps are required to access all other vertices from a given vertex. On the other hand, page rank approximates the probability of any message reaching a particular vertex. In the first group, there are 17 authors (blue cluster). Davis is the most prominent author (with a betweenness centrality value of 58.6, page rank of 0.04, and closeness of 0.01). The second group is made up of 16 authors (red cluster). Abbeduto stands out in it (with a centrality value of 32.1, page rank of 0.02, and closeness of 0.01). The third group is made up of 17 authors (green cluster). The main author on this group is Hayes (with a betweenness centrality value of 27.1, a page rank of 0.03, and a closeness value of 0.01) (see Figure 5).

### 3.7. Social Structure: Collaboration Network of Authors, Institutions, and Countries

**Figure 6:**
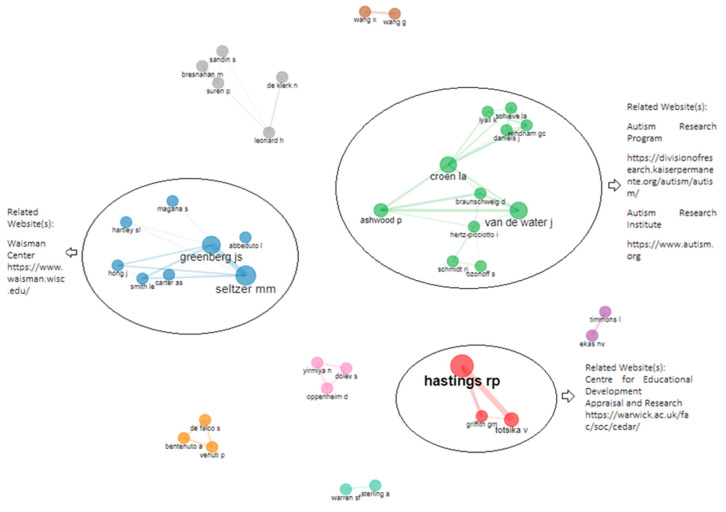
Collaboration network of the most representative authors in the field of maternity and ASD. Related websites: https://www.waisman.wisc.edu/ (Accessed on 4 April 2022), https://divisionofresearch.kaiserpermanente.org/autism/autism (Accessed on 4 April 2022), https://www.autism.org/ (Accessed on 4 April 2022) and https://warwick.ac.uk/fac/soc/cedar/ (Accessed on 4 April 2022).

Within the main author collaboration network, there are a total of 39 authors. Nine working groups can be observed in this network. The main working groups are visualized by means of the red cluster, consisting of Richard P. Hastings (lead author), Vasiliki Totsika, and Gemma M. Griffith; by the blue cluster, consisting of Marsha M. Seltzer (lead author), Jan S. Greenberg, Leonard Abbeduto, Jinkuk Hong, Sandy Magaña, Sigan L. Hartley, and Leann E. Smith; and by the green cluster, consisting of Lisa A. Croen (lead author), Judy Van de Water, Gayle C. Windham, Laura A. Schieve, Paul Ashwood, Daniel Braunschweig, Kristen Lyall, Julie L. Daniels, Irva Hertz-Picciotto, Sally Ozonoff, and Rebecca J. Schmidt (see Figure 6).

**Figure 7:**
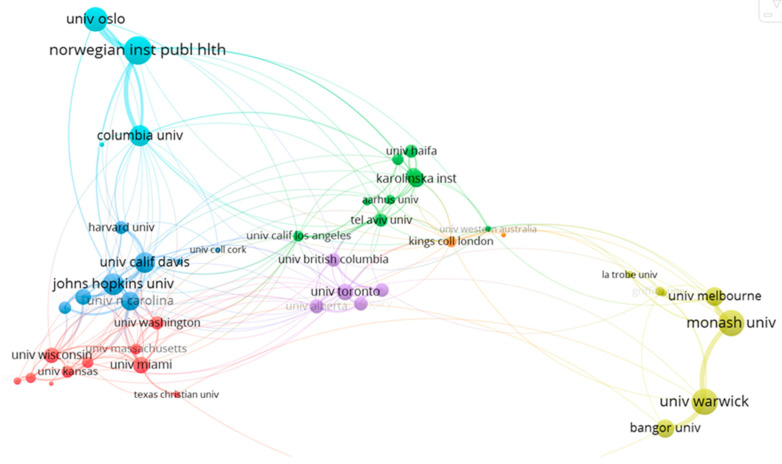
Collaboration network of the most representative institutions in the field of maternity and ASD.

A total of 1831 publications registered in WoS institutions are involved in the scientific production of maternity and ASD. In Figure 7, it can be seen that the main institutional collaboration networks are mainly grouped into six clusters. The most representative institutions from each cluster are the University of California (Davis) (n = 233 docs, dark blue cluster), the University of Wisconsin (n = 119 docs, red cluster), the University of Toronto (n = 36 docs, purple cluster), the Norwegian Institute of Public Health (n = 36 docs, light blue cluster), Monash University (n = 35 docs, yellow cluster) and the Karolinska Institutet (n = 30 docs, green cluster).

**Figure 8:**
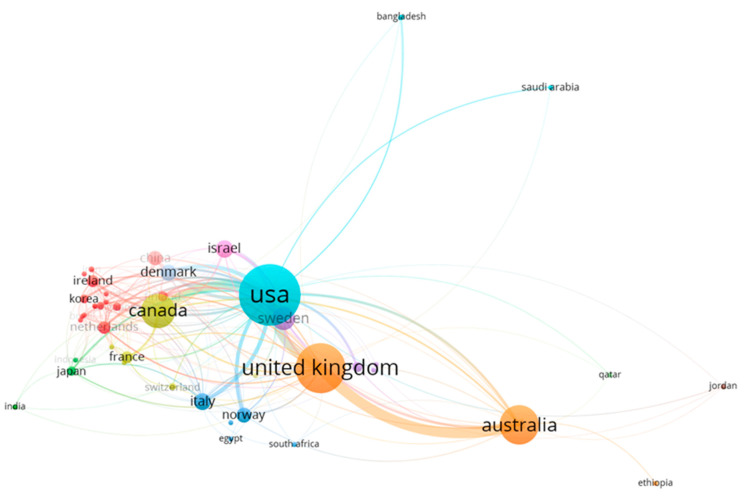
Collaboration network of the most representative countries in the field of maternity and ASD.

A total of 57 countries are involved in producing documents related to maternity and ASD. The USA (TC = 23,091; average article citations = 35.52) has the most significant weight in collaborations. Close collaboration can also be observed between the UK (TC = 4217; average article citations = 32.44) and Australia (TC = 2916; average article citations = 25.14), representing the second and third countries, respectively, with the most significant weight in collaborations (see Figure 8).

## 4. Discussion

The different analysis of this bibliometric study allows us to have a global and holistic understanding of the documents published in WoS related to motherhood and ASD. Document types such as original articles and documentary reviews (for example, systematic reviews and meta-analyses) are essential transmitters of relevant scientific information [48]. This is consistent with our findings in the field of maternity and ASD, since we found that the aforementioned types of documents represented 91% of the sample. The number of scientific documents in this field of study has grown exponentially in recent decades: from 2000 to 2010, 151 papers were published, but from 2010 to 2020, 1050 documents were published. This is most likely because, internationally, public health services for children diagnosed with ASD have grown considerably in recent years, leading to a substantial increase in the estimated prevalence of autism and a genuine interest in it. The most prolific and impactful authors were Richard P. Hastings (29 papers), Marsha M. Seltzer (21 papers), and Judy Van de Water (20 papers). The latest publications by these authors have covered a wide range of topics, such as educational elements during current public health events, the need of support programs for mothers, and the quality of the mother–child relationship that the child and mothers experienced as a possible autism spectrum risk biomarker. In terms of journals, *The Journal of Autism and Developmental Disorders* is the most prolific journal in the field of motherhood and ASD research. It is a multidisciplinary journal that seeks to promote theoretical and applied research examining and evaluating clinical diagnoses and treatments for ASD and related disabilities. The second most prolific journal was *Research in Autism Spectrum Disorders*, which is multidisciplinary in nature. It publishes high-quality empirical articles and reviews that contribute to a better understanding of autism spectrum disorders (ASD) at all levels of description: genetic, neurobiological, cognitive, and behavioral. The third most prolific journal was *Autism*, which is interdisciplinary and focuses on publishing research of direct relevance and practice to help to improve the quality of life of people with autism or autism-related disorders. These journals are at the forefront of ASD research and have also led other bibliometric reviews, for example, those by Carmona-Serrano et al. [49] and Feng et al. [50]. Regarding the most cited studies, they were published between 1997 and 2012.The study by Davis and Carter [34], “Parenting Stress in Mothers and Fathers of Toddlers with Autism Spectrum Disorders: Associations with Child Characteristics”, was the most cited among all those identified in this review. According to the criteria of Calderón et al. [51], the age of the articles is closely related to the number of citations received, which means that old articles that have dealt with a problem in a new way are more frequently cited and continue to receive citations over the years, since they serve as the basis and inspiration for further studies. There is evidence that stress remains a very topical issue for mothers of children with ASD [52,53]. Even very recently, a new line of research has been developed that addresses the variable of stress in mothers diagnosed with ASD [54] since, as a general rule, the literature had only focused on mothers of children and young people with ASD. To some extent, the validity and interest of the scientific community in this topic could explain why this document has been highly cited.

The analysis of the keywords indicates that the stress related to motherhood and the autism spectrum is a main and priority issue. The strength of the link between these themes is reflected in the keyword co-occurrence network (KCN). This is supported by a wide range of documentary studies. For example, a systematic review carried out by Ghanouni and Hood [55] highlights that the severity of the symptoms of the autistic child is one of the greatest influences on maternal well-being. Another integrative review of the literature, by Porter and Loveland [56], highlights the fact that mothers of children with ASD experience much greater parenting stress than mothers of typically developing children and children with other disabilities. The results of the thematic analysis also show that issues such as stress are emerging and connected to the parenting of children with ASD. However, this topic should not be limited only to the study of neurotypical mothers of children with autism spectrum and should also cover autistic mothers [54]. The study also showed that topics related to pregnancy, fetal brain, and antibodies are well established in the literature, all of which continue to present a challenge and important reasons for study to understand autism [57,58,59]. It is interesting to discover that topics such as prevalence, risk, and population tend to emerge and disappear with volatility in the trends in the literature studied. These findings provide a guiding visual image of the trends in this field of research, which can serve as a basis for the design of new studies.

The United States is the country with the highest number of contributions in the world, although the United Kingdom, Australia, and Canada also stand out. The most relevant institutions were the University of California (Davis), the Johns Hopkins University, Monash University, the Karolinska Institute, and the Norwegian Institute of Public Health. These countries and institutions have also been leaders in other bibliometric studies that have addressed research related to ASD [49,50,60,61]. These results suggest that developing countries, such as those in Latin America, should establish ties with Anglo-Saxon countries, since it could boost their development in this field of study. This would allow them to describe the local reality with new and cutting-edge scientific information related to motherhood and ASD.

## 5. Limitations and Recommendations

This work has some limitations that should be highlighted. The search was carried out only on the Web of Science, and although this database is one of the most important and comprehensive in the world, it does not, of course, contain all the publications in the field of motherhood research on the autism spectrum. Perhaps, for future studies, it would be convenient to use other international databases, such as Scopus. The bibliometric analysis is based on the general virtues of quantitative methods and, therefore, it is not possible to interpret the content or the quality of the publications selected as the object of study. Based on these limitations that are inherent to bibliometric analysis, it is recommended to use this technique in more particular issues of motherhood on the autism spectrum (for example, to examine the mental health of mothers of children on the autism spectrum). This can provide a more in-depth content analysis of certain variables and provide direction for future research.

## 6. Conclusions

In this study, we provide a global and holistic view of the research area related to motherhood on the autism spectrum. The most important countries come from Anglo-Saxon culture (USA, UK, Canada, and Australia). The United States of America, the United Kingdom, and Australia were the three most prolific countries and had the largest collaborative network in maternity and ASD research Nowadays, international cooperation continues to be practically nil in Latin American countries. Developing countries need to link up with Anglo-Saxon countries so that they can draw on research experience through international cooperation. Variables related to mental health (for example, stress) turned out to be clear trends today. The main studies that have served as guides come from different disciplinary fields, highlighting the need for interdisciplinary research to address ASD-related childbearing. This study provides a picture of the current state of research on motherhood in ASD, based on WoS. In addition to this, due to its bibliometric nature, it can provide useful information for new researchers to find possible collaborators and guidance for designing studies that provide valuable information.

## Figures and Tables

**Table 1:** The number of annual publications related to motherhood research on the autism spectrum from 1976 to 2021.

First Period (6 Documents)		Second Period (151 Documents)		Third Period (1050 Documents)		Fourth Period (387 Documents)	
Year	Articles	Year	Articles	Year	Articles	Year	Articles
1990	0	2000	4	2010	54	2020	183
1991	1	2001	6	2011	58	2021	183
1992	0	2002	5	2012	55	2022	21
1993	0	2003	9	2013	88		
1994	0	2004	13	2014	103		
1995	2	2005	10	2015	100		
1996	0	2006	15	2016	137		
1997	1	2007	24	2017	147		
1998	2	2008	33	2018	163		
1999	0	2009	36	2019	165		

**Table 2:** Most prolific authors.

Authors	Articles (n)	Articles Fractionalized	h Index	g Index	m Index	TC	NP	PY Start
Hastings, R.P.	29	8.24	16	29	0.008	1772	29	1994
Seltzer, M.M.	21	5.97	17	18	0.895	1607	18	2004
Van de water, J.	20	4.15	15	19	1.000	1168	19	2008
Greenberg, J.S.	18	4.75	14	18	0.737	1110	18	2004
Ekas, N.V.	16	4.95	11	16	0.786	522	16	2009
Totsika, V.	16	4.28	8	14	0.667	488	14	2011
Croen, L.A.	15	1.98	10	15	0.476	1048	15	2002
Ashwood, P.	13	1.82	12	13	0.800	1113	13	2008
Benson, P.R.	13	11.75	9	11	0.529	544	11	2006
Abbeduto, L.	12	3.21	6	9	0.316	618	9	2004

Note: Articles fractionalized (Fractional authorship quantifies an individual author’s contributions to a published set of papers); h-index (author-level metric that attempts to measure both the productivity and citation impact of the publications of a scientist or scholar; g -index (quantifies the bibliometric productivity based on the publication history of authors); m index (this value represents the average amount the author’s h-index has increased per year over his or her publishing career and can help differentiate between two authors with similar h-indexes but different career lengths); TC (total citations; the growth rate shows the productive increase; it is the percentage difference of the number of jobs in relation to the previous period); NP (number of publications); PY (year in which publication began).

**Table 3:** Lotka’s law, to calculate the correlation of the number of authors with the published articles.

Documents Written	Number of Authors	Proportion of Authors
1	4509	0.839
2	545	0.101
3	165	0.031
4	68	0.013
5	29	0.005
6	20	0.004
7	18	0.003
8	6	0.001
9	5	0.001
10	1	0
12	1	0
13	2	0
15	1	0
16	2	0
18	1	0
20	1	0
21	1	0
29	1	0

**Table 4:** Top 10 journals that have published articles on motherhood and ASD.

Sources	Articles (n)	h_Index	g_Index	m_Index	TC	NP	PY_Start	Country
*The Journal of Autism and Developmental Disorders*	169	45	79	0.022	6901	158	1999	USA
*Research in Autism Spectrum Disorders*	65	17	31	1.308	1127	55	2010	NL
*Autism*	57	24	53	0.012	2851	56	1999	UK
*Research in Developmental Disabilities*	43	15	26	1.071	724	39	2009	USA
*Autism Research*	40	17	28	1.308	826	37	2010	USA
*Journal of Intellectual Disability Research*	29	12	20	0.429	1346	20	1995	UK
*Journal of Child and Family Studies*	25	9	20	0.692	425	21	2010	USA
*Journal of Applied Research in Intellectual Disabilities*	20	10	16	0.625	429	16	2007	UK
*Journal of Intellectual Developmental Disability*	19	8	18	0.421	404	18	2004	UK
*Journal of Developmental and Physical Disabilities*	18	8	16	0.381	267	17	2002	USA

Note: h-index is determined by sorting all the publications by a person in descending order of their citation frequency. The value where the sequential number of the publication tallies with the citation frequency is the Hirsch factor.

**Table 5:** The most prominent grassroots journals in maternity and ASD research, based on Bradford’s law.

Sources	Rank	Freq	Cum Freq	Zone
*The Journal of Autism and Developmental Disorders*	1	169	169	Zone 1
*Research in Autism Spectrum Disorders*	2	65	234	Zone 1
*Autism*	3	57	291	Zone 1
*Research in Developmental Disabilities*	4	43	334	Zone 1
*Autism Research*	5	40	374	Zone 1
*Journal of Intellectual Disability Research*	6	29	403	Zone 1
*Journal of Child and Family Studies*	7	25	428	Zone 1
*Journal of Applied Research in Intellectual Disabilities*	8	20	448	Zone 1
*Journal of* *Intellectual and Developmental Disability*	9	19	467	Zone 1
*Journal of Developmental and Physical Disabilities*	10	18	485	Zone 1
*Brain Behavior and Immunity*	11	16	501	Zone 1
*International Journal of Developmental Disabilities*	12	15	516	Zone 1
*Plos One*	13	14	530	Zone 1
*Journal of Child Psychology and Psychiatry*	14	13	543	Zone 1
*Disability & Society*	15	12	555	Zone 1

Note: Freq: frequency. Cum Freq: cumulative frequency distribution, the sum of the class and all classes below it in frequency distribution. Thus, the result of the sum of a value and all of the values that came before it. Zone: for any single issue, or subject area, the top third (Zone 1 or Core) represents the journals that are the most frequently cited in the literature of that subject and that are, therefore, likely to be of highest interest to researchers in the discipline.

**Table 6:** The characteristics and main results of highly cited papers on research related to maternity and ASD.

Reference	Journal	Title	Total Citations	TC per Year	Normalized TC	Main Result
Davis and Carter (2008) [35]	*The Journal of Autism and Developmental Disorders*	“Parenting Stress in Mothers and Fathers of Toddlers with Autism”	601	40.07	9.29	Parents reported elevated parenting stress. Deficits/delays in children’s social relatedness were associated with overall parenting stress, parent–child relationship problems, and distress for mothers and fathers.
Cook et al. (1997) [36]	*American Journal of Human Genetics*	”Autism or atypical autism in maternally but not paternally derived proximal 15q duplication”	479	18.42	1.00	During the course of the genotyping of trios of affected probands with AD and their parents, at the positional candidate locus D15S122, an intrachromosomal duplication of proximal 15q was detected by microsatellite analysis in a phenotypically normal mother.
Abbeduto et al. (2004) [38]	*American Journal on Mental Retardation*	”Psychological Well-Being and Coping in Mothers of Youths With Autism, Down Syndrome, or Fragile X Syndrome”	472	24.84	3.31	Mothers of individuals with fragile X syndrome displayed lower levels of well-being than those of individuals with Down syndrome, but higher levels than mothers of individuals with autism, although group differences varied somewhat across different dimensions of well-being. The most consistent predictor of maternal outcomes was the adolescent or young adult’s behavioral symptoms.
Estes et al. (2009) [39]	*Autism*	”Parenting stress and psychological functioning among mothers of preschool children with autism and developmental delay”	431	30.79	6.57	Evidence for higher levels of parenting stress and psychological distress was found in mothers in the ASD group compared to the DD group. Children’s problem behavior was associated with increased parenting stress and psychological distress in mothers in the ASD and DD groups.
Dabrowska, and Pisula, (2010) [40]	*Journal of Intellectual Disability Research*	”Parenting stress and coping styles in mothers”	386	29.69	6.49	The results indicated a higher level of stress in parents of children with autism. Mothers of children with autism scored higher than fathers in parental stress.
Gardener et al. (2009) [41]	*The British Journal of Psychiatry*	”Prenatal risk factors for autism: comprehensive meta-analysis”	382	27.29	5.83	Over 50 prenatal factors were examined. The factors associated with autism risk in the meta-analysis were advanced parental age at birth, maternal prenatal medication use, bleeding, gestational diabetes, being first born vs. third or later, and having a mother born abroad. The factors with the strongest evidence against a role in autism risk included previous fetal loss and maternal hypertension, proteinuria, pre-eclampsia and swelling.
Malkova et al. (2012) [37]	*Brain, Behavior, and Immunity*	”Maternal immune activation yields offspring displaying mouse versions of the three core symptoms of autism”	375	34.09	7.09	These results indicate that MIA yields male offspring with deficient social and communicative behavior, as well as high levels of repetitive behaviors, all of which are hallmarks of autism.
Herring et al. (2006) [42]	*Journal of Intellectual Disability Research*	”Behaviour and emotional problems in toddlers with pervasive developmental disorders and developmental delay: associations with parental mental health and family functioning”	350	20.59	3.53	Initial and follow-up measures of child behavior and emotional problems, parent mental health problems, parent stress, and family functioning were significantly correlated, providing some evidence of stability over time. Child emotional and behavioral problems contributed significantly more to mother stress, parent mental health problems, and perceived family dysfunction than child diagnosis (PDD/non-PDD), delay, or gender.
Hastings et al. (2005) [43]	*Journal of Autism and Developmental Disorders*	”Systems Analysis of Stress and Positive Perceptions in Mothers and Fathers of Pre-School Children with Autism”	345	19.17	2.18	Mothers were found to report both more depression and more positive perceptions than fathers. Regression analyses revealed that paternal stress and positive perceptions were predicted by maternal depression; maternal stress was predicted by their children’s behavior problems (not adaptive behavior or autism symptoms) and by their partner’s depression.
Glasson et al. (2004) [44]	*Archives of General Psychiatry*	”Perinatal Factors and the Development of Autism”	345	18.16	2.42	Case mothers had greater frequencies of threatened abortion, epidural caudal anesthesia use, labor induction, and a labor duration of less than 1 h. Cases were more likely to have experienced fetal distress, been delivered by an elective or emergency cesarean section, and had an Apgar score of less than 6 at 1 min. Cases with a diagnosis of autism had more complications than those with pervasive developmental disorder not otherwise specified or Asperger syndrome.

Note: total citations (TC); normalized TC (the normalized TC is calculated to provide equal credit of citation to all the authors of the paper).

## Data Availability

The data presented in this study are openly available in Zenodo at https://doi.org/10.5281/zenodo.7613091 (accessed on 27 January 2023).
